# Supplementary figures and images for: Small molecule allosteric inhibitors of RORγt block Th17-dependent inflammation and associated gene expression in vivo
Source: PLoS One. 2021 Nov 9;16(11):e0248034. doi: 10.1371/journal.pone.0248034 (PMC8577775; doi:10.1371/journal.pone.0248034)

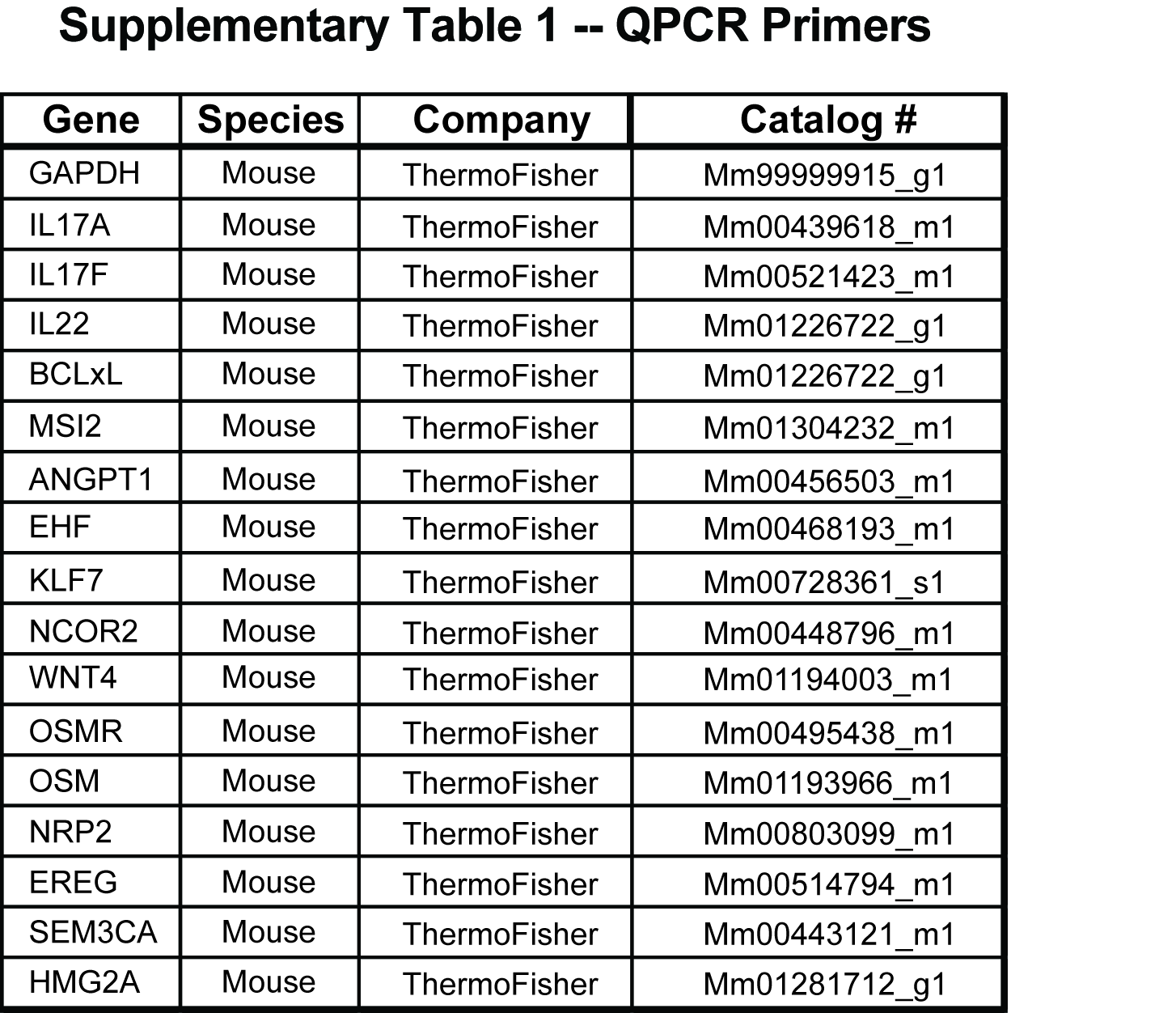

Supplement: S1 Table — (TIF) [file pone.0248034.s001.tif]

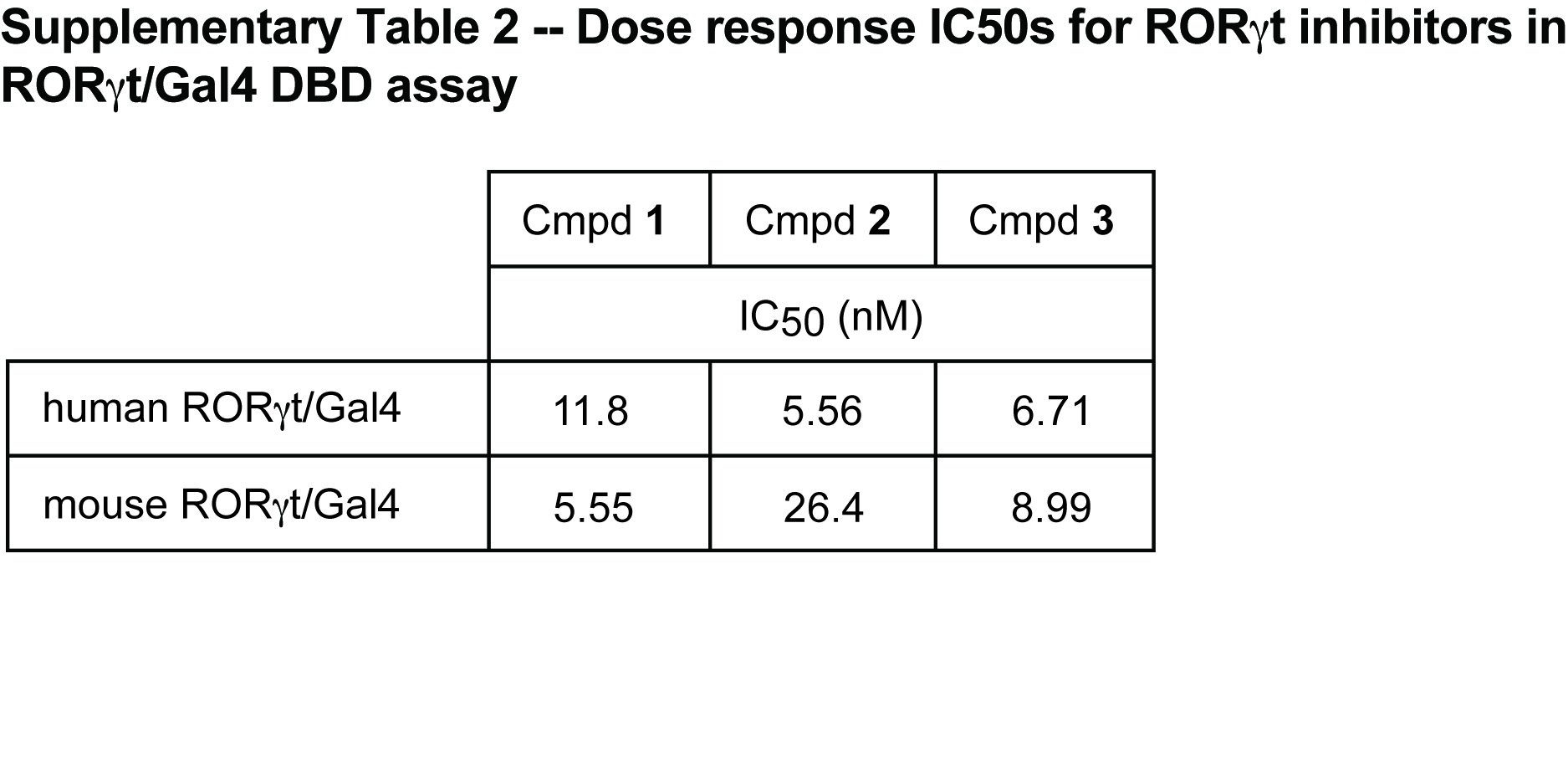

Supplement: S2 Table — Concentrations of the RORγt inhibitors, Compound 1, Compound 2 or Compound 3, in which 50% (IC50) relative light units (RLUs) was inhibited. Assays utilized reporter cells (HEK293) harboring a receptor hybrid in which the native N-terminal DNA binding domain (DBD) has been replaced with that of the yeast Gal4 DBD, with a firefly luciferase reporter gene functionally linked to a Gal4 upstream activation sequence. (TIF) [file pone.0248034.s002.tif]

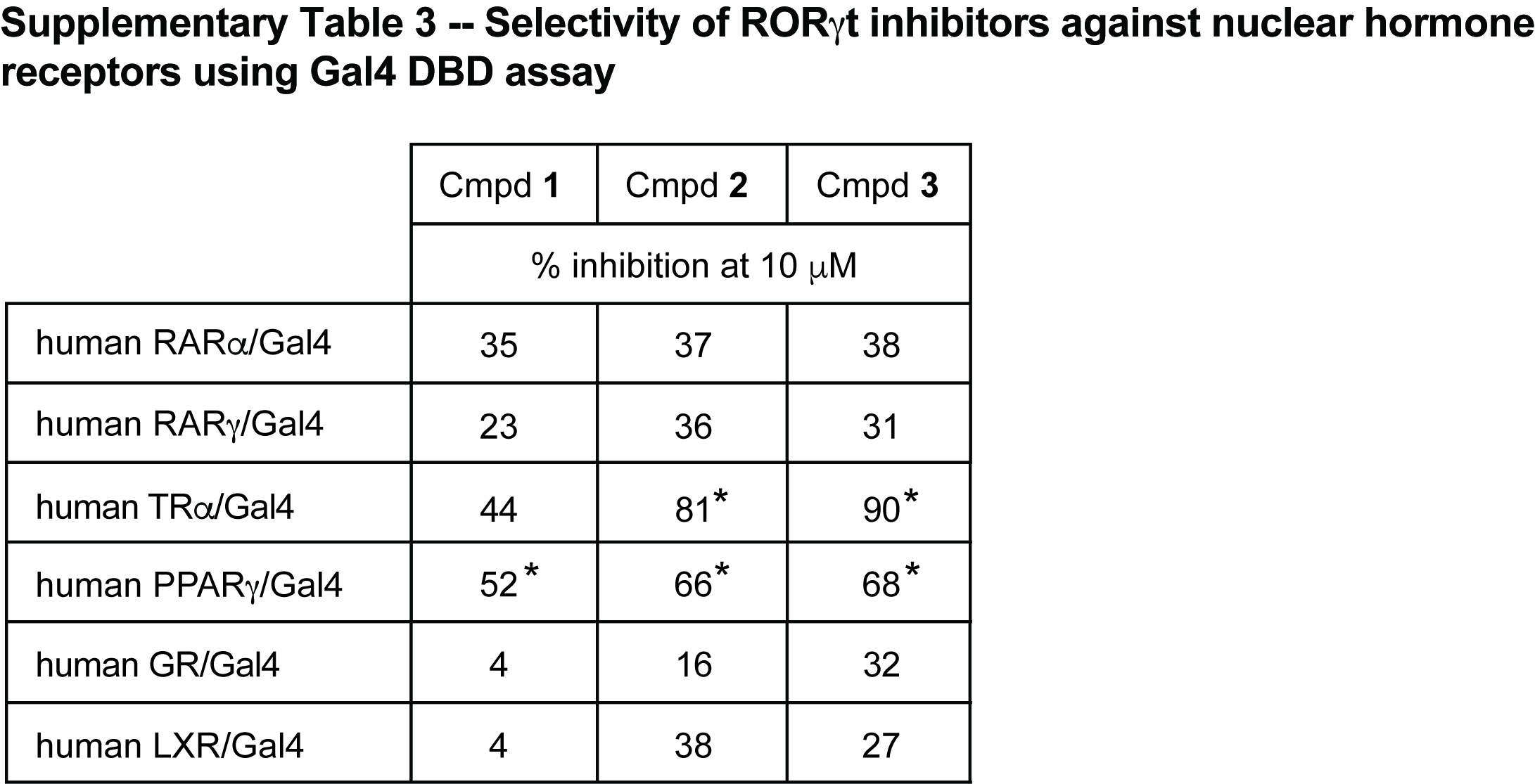

Supplement: S3 Table — RORγt inhibitor selectivity of Compound 1, Compound 2 or Compound 3 was assessed in nuclear hormone receptor binding assays, as measured by percent inhibition. Assays utilized reporter cells (HEK293) harboring a receptor hybrid in which the native N-terminal DNA binding domain (DBD) has been replaced with that of the yeast Gal4 DBD, with a firefly luciferase reporter gene functionally linked to a Gal4 upstream activation sequence. All compounds were dosed at 10 μM and percent inhibition calculated by relative light units (RLUs) compared to vehicle. *, repeat dose response curves failed to generate reliable IC50 values for compounds tested. RAR, RAR-related orphan receptor alpha; PPARγ, peroxisome proliferator-activated receptor gamma; TRα, thyroid hormone receptor alpha; GR, glucocorticoid receptor; LXR, liver X receptor. (TIF) [file pone.0248034.s003.tif]

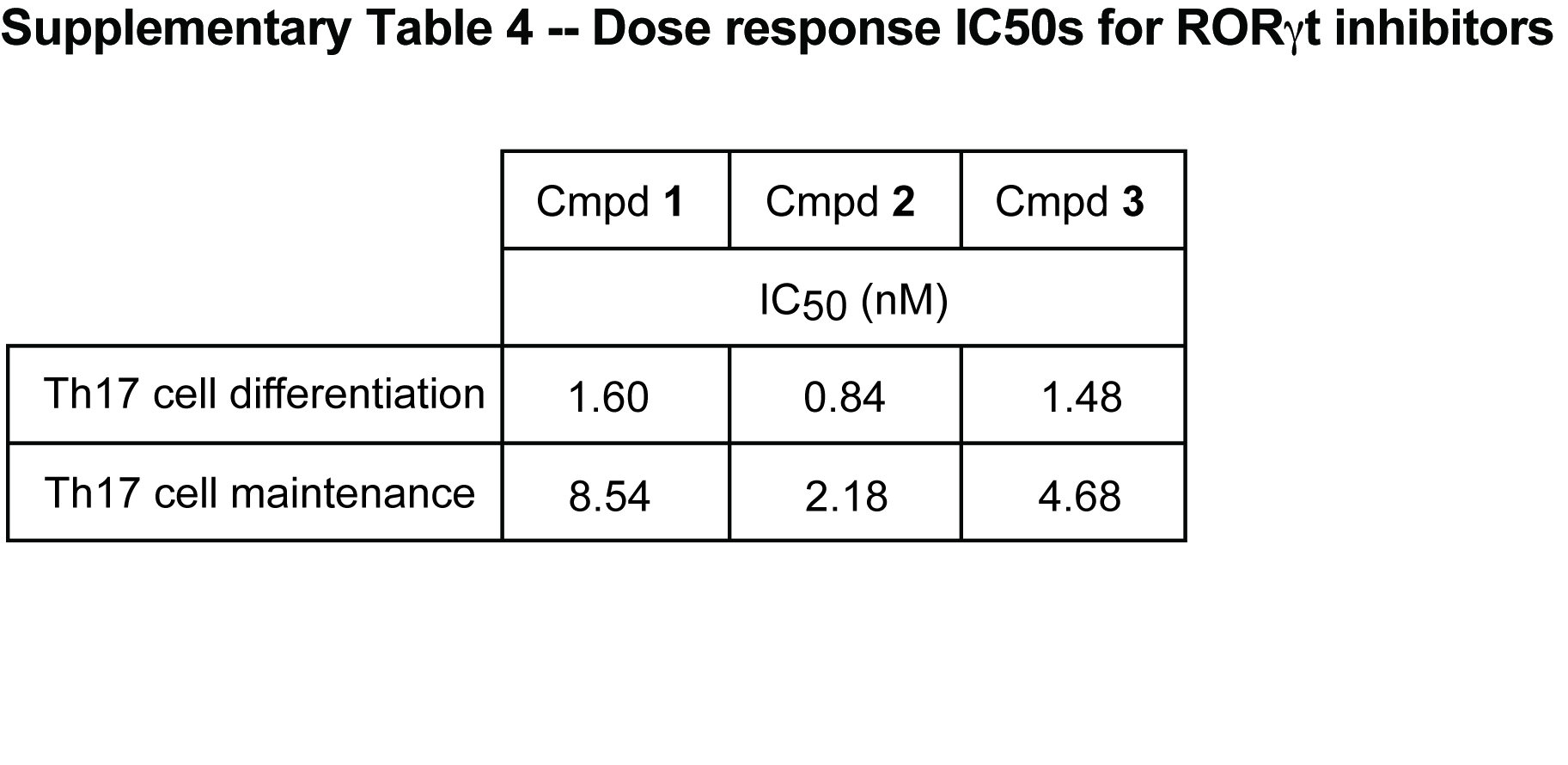

Supplement: S4 Table — Concentrations of RORγt inhibitors Compound 1, Compound 2 or Compound 3 in which 50% (IC50) IL-17A cytokine was inhibited in Th17 differentiation or Th17 maintenance assays. (TIF) [file pone.0248034.s004.tif]

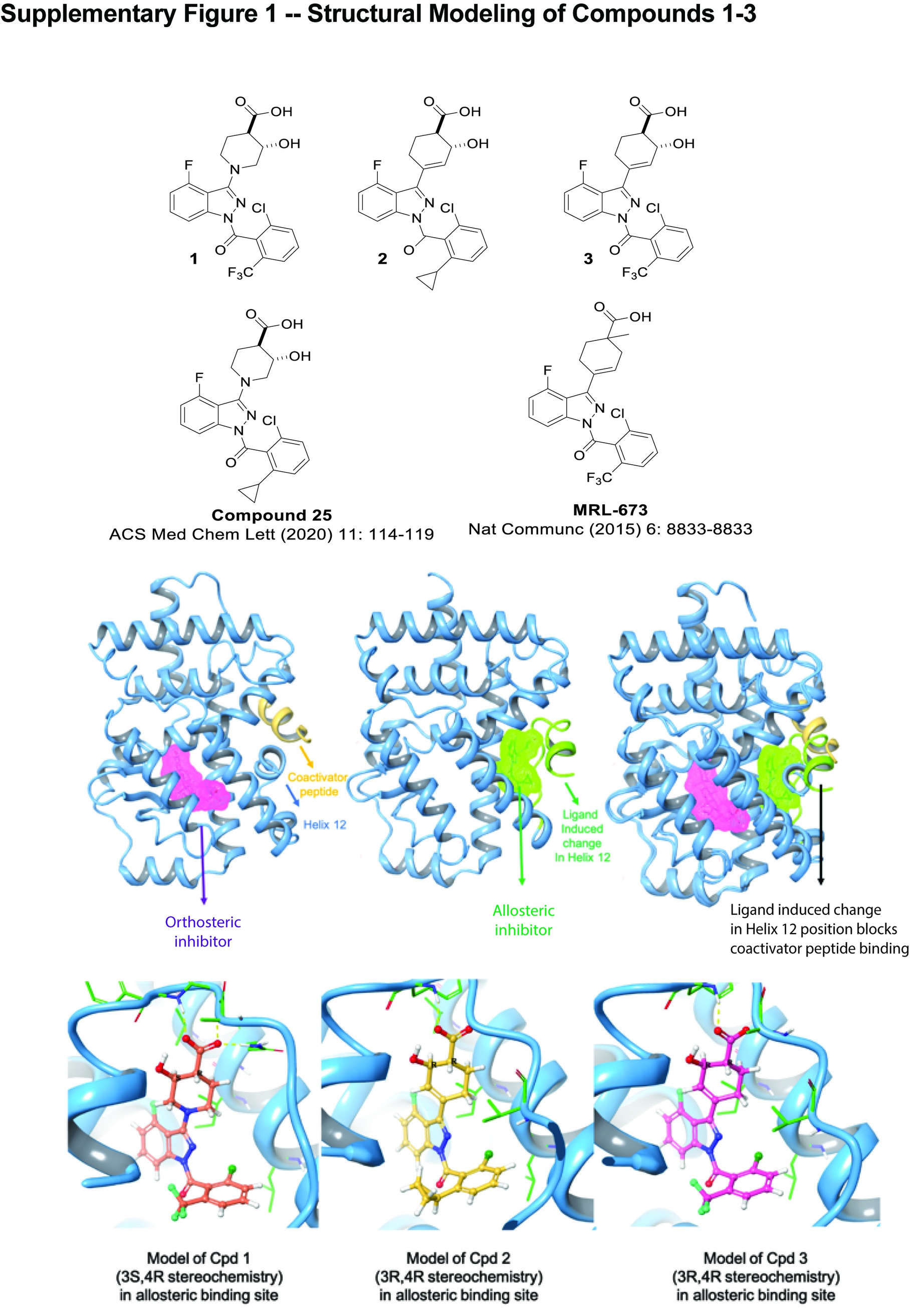

Supplement: S1 Fig — Structural models of compounds 1–3 were built in RORγt structures (5C4T, 6UCG). The crystal structures were imported into Maestro (Schrödinger Release 2020–2: Maestro, Glide, LigPrep, Epik, Schrödinger, LLC, New York, NY, 2020.). The structures were prepared using the Protein Preparation workflow as implemented in the Schrödinger Suite. Glide docking grids were generated by focusing the grid box on the center of the Cpd25 and MRL-673. The size of the box enclosing the grid was set to 10 Å. No other constraints, rotatable groups or excluded volumes were imposed. The three compounds 1–3 were then prepared for docking using LigPrep and the OPLS3e force field was used for minimizations; possible ionization states at pH 7.0 ± 2.0 were generated using Epik and tautomers were generated; specified chirality was retained. (TIF) [file pone.0248034.s005.tif]

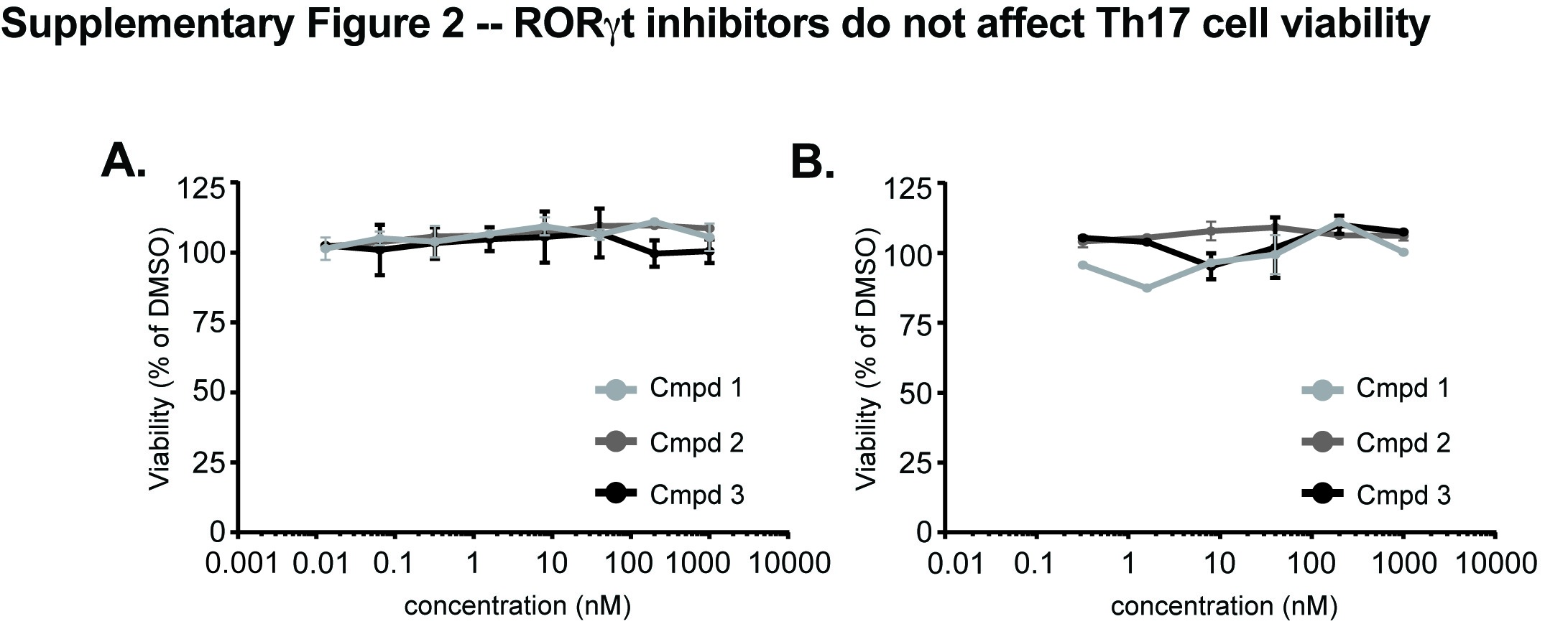

Supplement: S2 Fig — Cell viability of enriched naïve CD4+ T cells from healthy donor PBMC cultured under Th17 cell conditions, for 6 days (differentiation) (A) or enriched human Th17 cells from healthy donor PBMC cultured with IL-23 and IL-1β, for 4 days (maintenance) (B) in the presence of RORγt inhibitors Compound 1, Compound 2 or Compound 3. Data are normalized to and represented as percent of DMSO control. Error bars are representative of 4 individual donors, from 2 independent experiments. (TIF) [file pone.0248034.s006.tif]

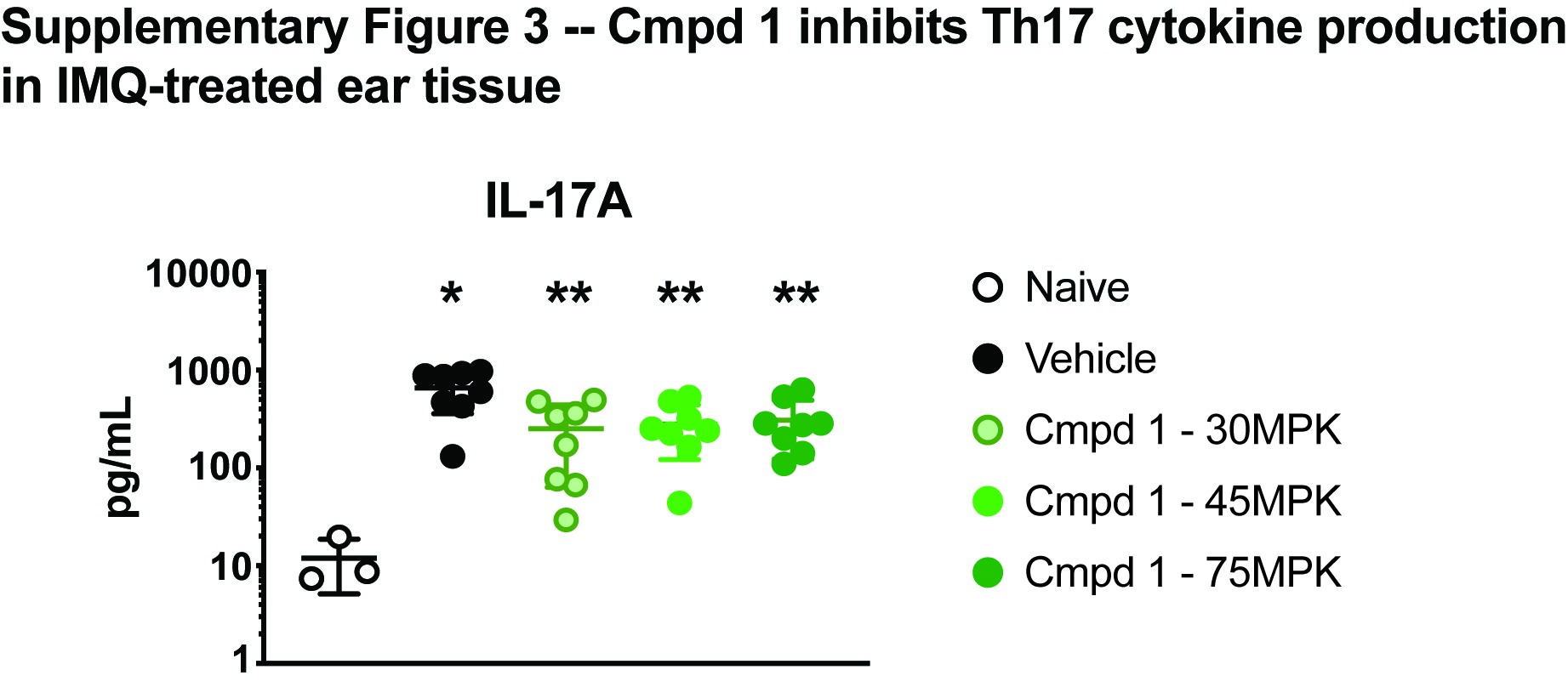

Supplement: S3 Fig — IL-17A cytokine levels were measured by Luminex assay from the supernatants of ear tissue ‘floats’ cultured for 24 hours ex vivo. Each symbol represents an individual animal and error bars denote mean ± SEM. Statistical significance (*p ≤ 0.05) was determined using one-way ANOVA with Tukey’s multiple comparisons test, *significant over naïve; **significance over vehicle-treated group. Data are representative of 2 independent experiments with n = 3-8/group. (TIF) [file pone.0248034.s007.tif]

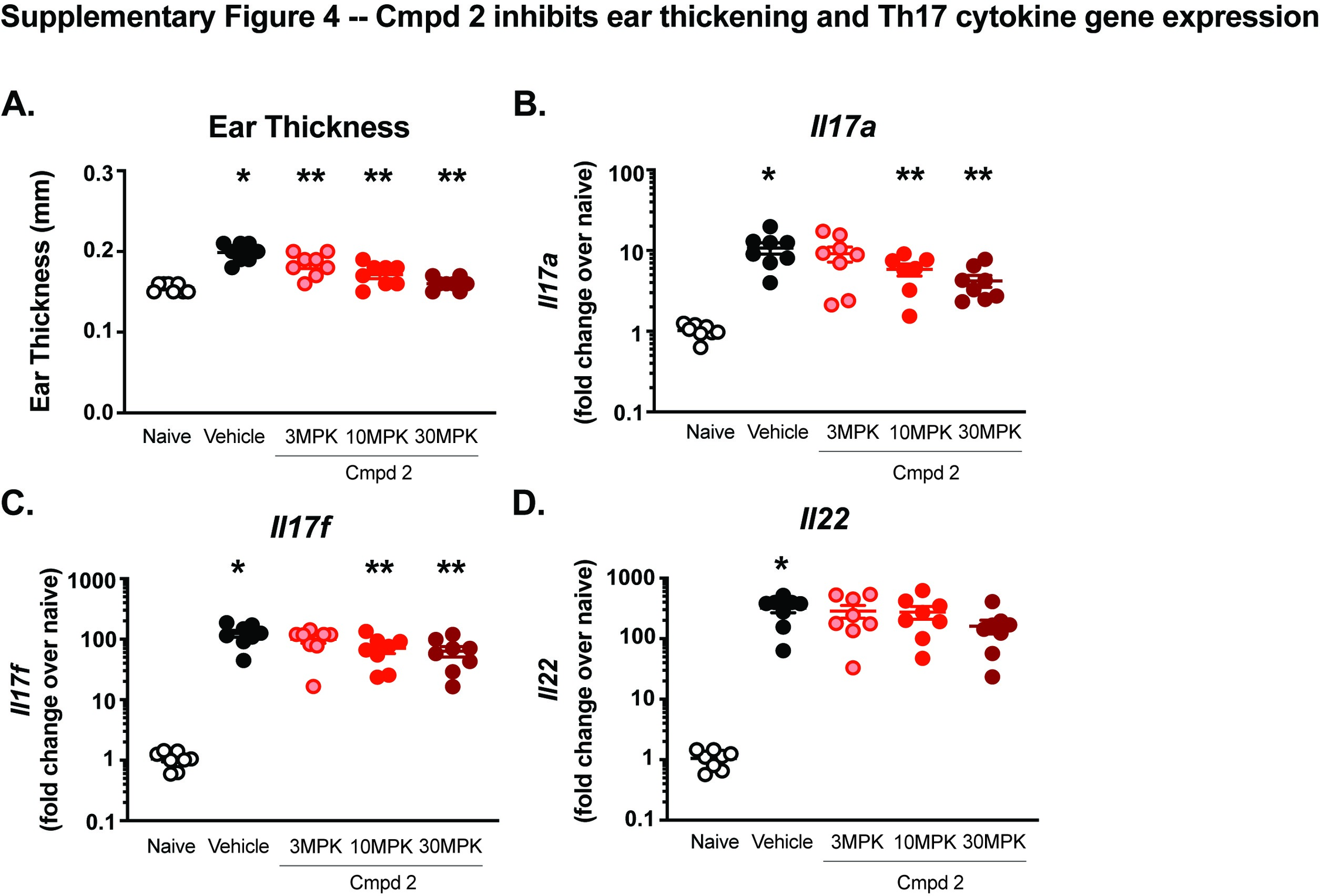

Supplement: S4 Fig — Ear thickness (mm) was measured in naïve or IMQ-treated animals on day 4 using digital micro-calipers (A). Th17 cytokine gene expression analysis was performed for Il17a (B), Il17f (C) and Bclxl (D) on day 4. Expression is normalized to Gapdh and presented as fold change over naïve. Each symbol represents an individual animal and error bars denote mean ± SEM. Statistical significance (*p ≤ 0.05) was determined using one-way ANOVA with Tukey’s multiple comparisons test, *significant over naïve; **significance over vehicle-treated group. Data are representative of 2 independent experiments with n = 8/group. (TIF) [file pone.0248034.s008.tif]

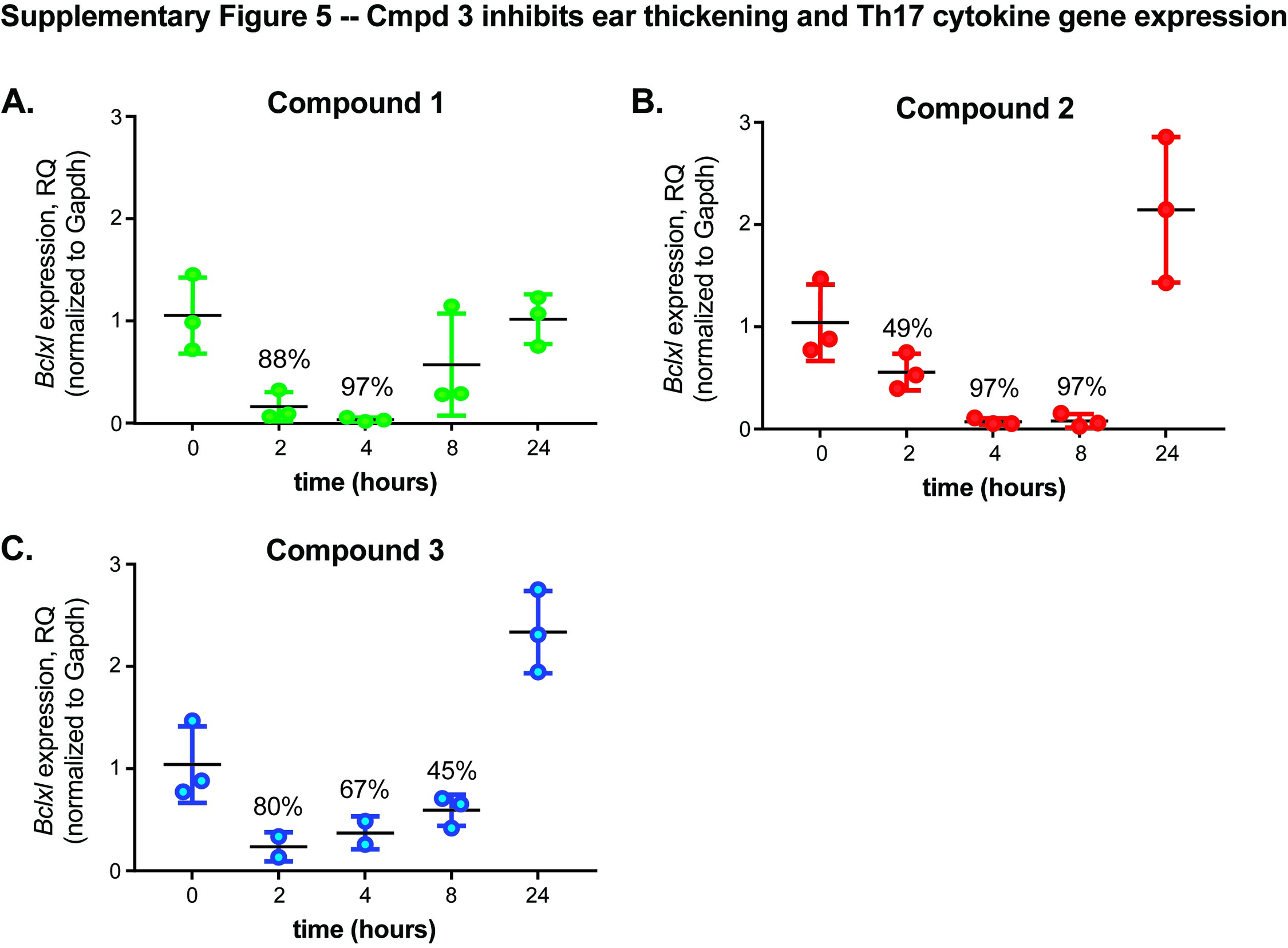

Supplement: S5 Fig — C57Bl/6 female mice were dosed PO with 100 mg/kg Compound 1, 2 or 3. Thymic tissues were collected from separate cohorts at 2, 8, 16 and 24 hours post-dose (n = 3 mice/timepoint). RNA was extracted from thymic tissues and Bclxl expression measured by QPCR. Data are normalized to housekeeping gene (Gapdh) and displayed as relative quantification. Time zero (‘0’) used as normalization timepoint and set to 1.0 RQ and mean set at 100% for % inhibition calculation. One-way ANOVA with Bonferroni correction for multiple comparisons used for statistical significance calculations. (TIF) [file pone.0248034.s009.tif]

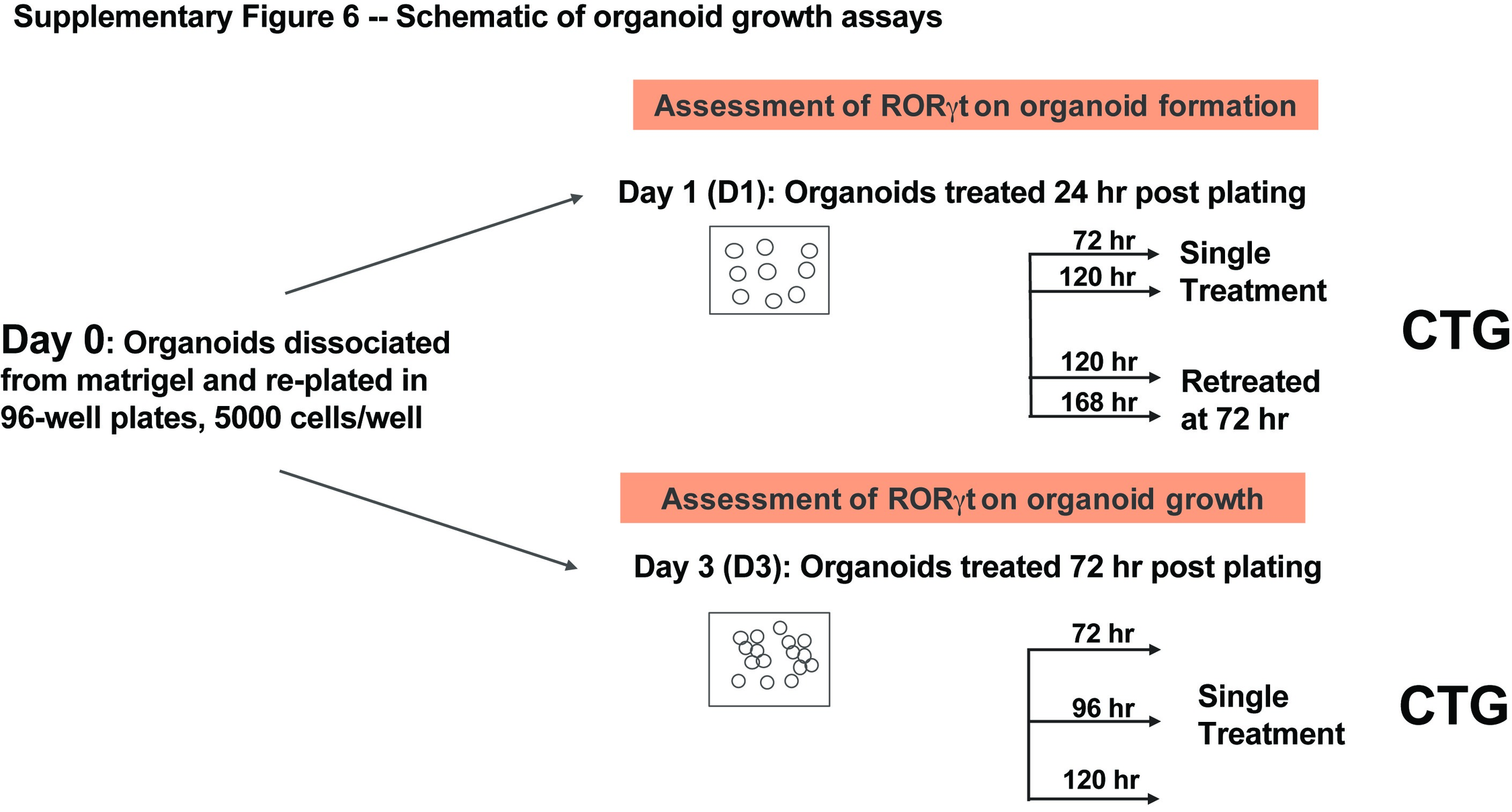

Supplement: S6 Fig — PDOs were dissociated into single cells and plated on day 0. Organoids were treated beginning on day 1 or day 3 to assess effect on organoid formation or organoid growth, respectively. After the indicated treatment schedule organoid growth or formation was assessed by CTG assay. (TIF) [file pone.0248034.s010.tif]

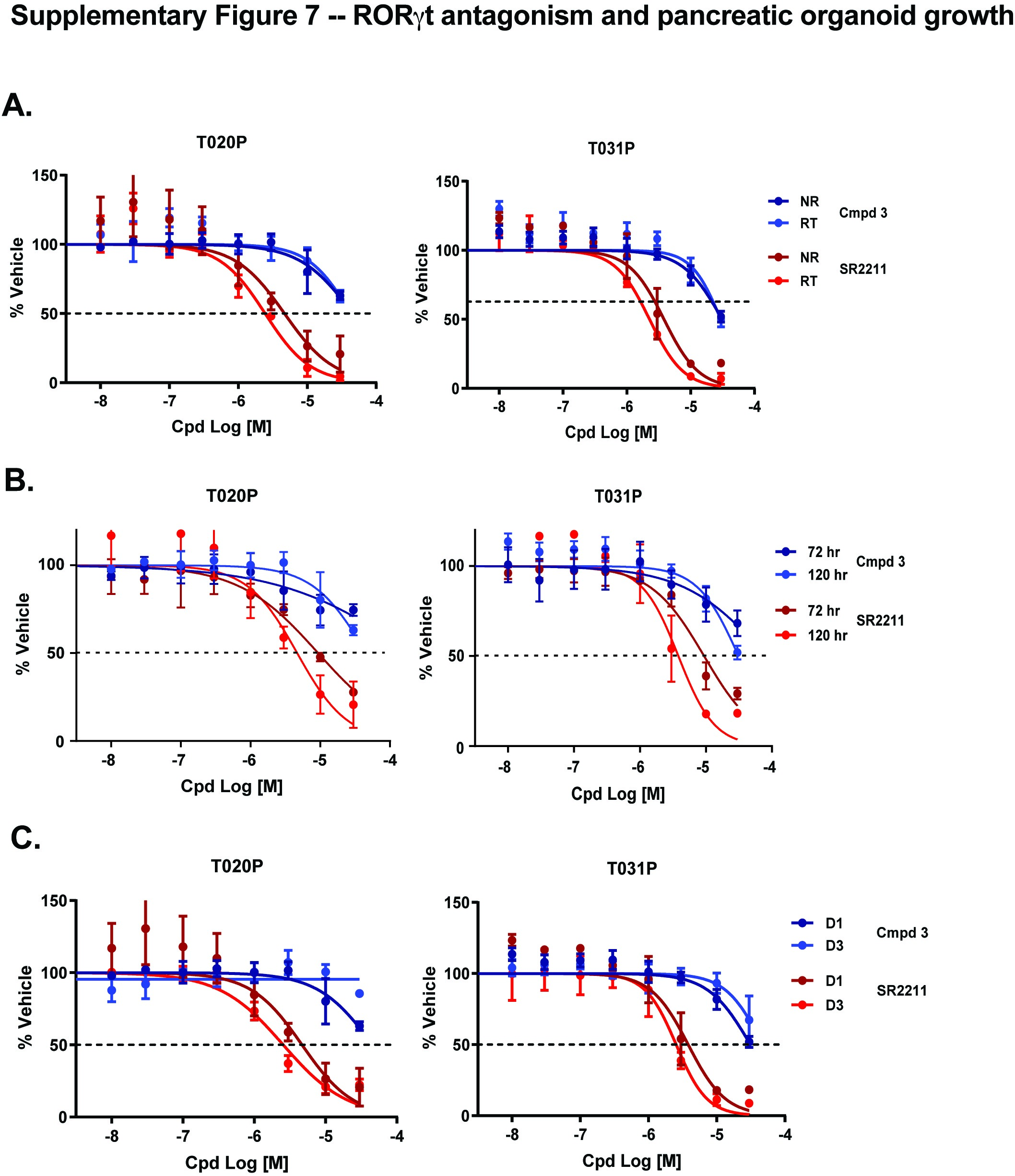

Supplement: S7 Fig — (A) PDOs were treated with Compound 3 or SR221 for 120 hours starting on day 1 after plating. PDOs were either subjected to a single treatment (NR) or compounds were replenished after 72 hours (RT). All values were calculated as % of vehicle treated organoids in 3 replicate experiments. (B) PDOs were treated with either Compound 3 or SR2211 for 72 or 120 hours starting on day 1 after plating. All values calculated as % vehicle treated organoids in 3 replicate experiments. (C) PDOs were treated for 120 hours with Compound 3 or SR2211 beginning on day 1 (D1) or day 3 (D3) post-plating. All values were calculated as % of vehicle treated organoids in 3 replicate experiments. (TIF) [file pone.0248034.s011.tif]

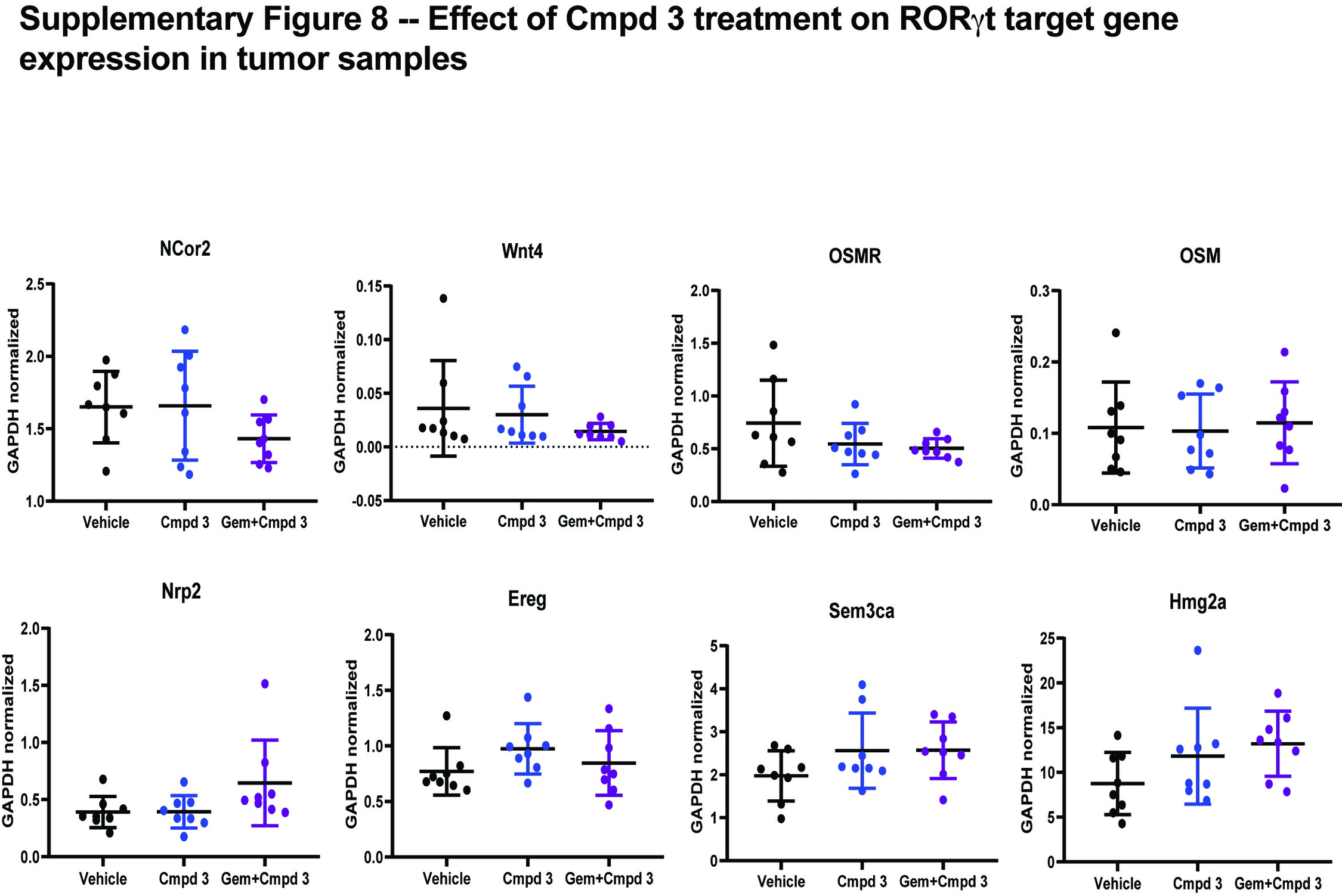

Supplement: S8 Fig — qPCR expression analysis of indicated genes in vehicle, Compound 3 or Compound 3 plus Gemcitabine treated tumor samples. Replicate values plotted individually with mean ± SD represented. (TIF) [file pone.0248034.s012.tif]
